# A latch on priming

**DOI:** 10.3389/fpsyg.2014.00869

**Published:** 2014-08-11

**Authors:** Alberto Bernacchia, Giancarlo La Camera, Frédéric Lavigne

**Affiliations:** ^1^School of Engineering and Science, Jacobs University Bremen gGmbHBremen, Germany; ^2^Department of Neurobiology and Behavior and Program in Neuroscience, State University of New York at Stony BrookStony Brook, NY, USA; ^3^Laboratoire Bases, Corpus, Langage, UMR 7320 CNRS, Université de Nice - Sophia AntipolisNice, France

**Keywords:** coding, model, mixed selectivity, neural network, noise, reinforcement learning, semantic, working memory

Semantic priming is the phenomenon for which presenting a word (“prime”) to a subject can influence the processing of a second word (“target”), such as in the “lexical decision task” (is the target a word or a non-word?) or in the out-loud pronunciation of the target word (“naming task”). Performance and response times are affected by the strength of the prime-target association (for example, “dog” and “cat” are more strongly associated than “dog” and “goat”), the delay between prime and target, and other factors (McNamara, 2005). Lerner and Shriki (2014) review and provide novel evidence for a model of semantic priming that accounts for many of the empirical findings and makes clear predictions on two new experiments conducted by the authors.

The Lerner and Shriki model combines the idea of distributed coding of concepts (Masson, 1991, 1995; Plaut, 1995) with the idea of “latching dynamics” in neural networks possessing attractor states—i.e., stable distributed activation patterns across units (Treves, 2005; Moreno-Bote et al., 2007). In attractor networks, correlated attractors that share units in the same activation states code for semantically related concepts. These networks generate priming effects by, e.g., speeding up the convergence to the attractor state coding for a target when the latter shares units with the prime. The ingredient added by Lerner and Shriki to this modeling framework is that their network is in an ever present dynamical regime: the state of the network does not dwell on a given attractor concept for long and tends to jump between attractors due to short-term synaptic depression. In their modeling approach, the authors explore a number of interesting ideas in the context of semantic priming. In doing so, they facilitate the convergence of the dominant connectionist style of modeling toward a neurobiological understanding of semantic priming.

First, by introducing latching dynamics between related concepts—one cued by the prime, the other leading to the target—their model embraces the idea that neural representations may be understood not only in terms of static patterns of neural activation, but also in terms of temporal dynamics of those patterns, where the dynamics can be adjusted to make correct (or faster) decisions. Recent evidence suggests that cognitive functions unfold on a variety of timescales, and so does the underlying neural activity. For example, working memory is dynamic: memorized items are intrinsically unstable and they may jump in and out of memory (Amit et al., 2003). The process of making a sensorimotor decision is also dynamic, being mediated by the dynamical integration of sensory evidence by neurons in parietal and prefrontal cortex (Wang, 2008). This applies to fast decisions (of the order of hundreds of milliseconds) based on immediate sensory evidence as well as to slower decisions (order of minutes) requiring careful evaluation of previous decisions and outcomes (Bernacchia et al., 2011). Finally, even after a decision is made, the production of the actual movement by motor cortical neurons seems best explained in the framework of dynamical systems, rather than from the more classical view of static population coding (Shenoy et al., 2013). The work of Lerner and Shriki fits into these lines of evidence and adds semantic priming to the repertoire of phenomena that can be explained in terms of the complex dynamic behavior of ensembles of cortical neurons.

Second, the authors introduce a role for intrinsically generated noise on priming effects in an original application of reinforcement learning. Well established in cortical models of decision-making (Wang, 2008) and reinforcement learning (where it allows exploration—see, e.g., Sutton and Barto, 1998), the role of neural noise in semantic priming has not been explored. In an interesting development, the authors introduce the idea of learning the intrinsic level of noise by reinforcing faster reaction times, so as to match the latter's strong empirical dependence on expectancy and other types of context. The authors consider this as the potential mechanism underlying controlled processing in semantic priming. Controlled processes are those modifiable by task demands (whereas automatic processes reflect the static associations between targets and primes). A more biological way of implementing this idea would be to perturb the membrane conductances of spiking neurons and thus change the background fluctuations in the network (Chance et al., 2002). This would change the response of a population of neurons by acting on the variance of the ongoing activity, rather than on the average input, in the presence of a priming cue. By “learning the variance rather than the mean,” the system would learn to respond differently in different contexts of the same task, rather than learning to perform the task itself—as is more customary. The feasibility of this approach should of course be tested in a detailed spiking neuron implementation.

Third, the Lerner and Shriki model shares some notable features with network models of working memory, bringing the connectionist framework closer to more biologically plausible cortical models. Following the connectionist tradition, however, in the Lerner and Shriki model the associations are generated *ad hoc* to define which pairs of concepts will generate priming effects. In cortical models, priming effects depend on learned values of synaptic strength between neurons coding for the more strongly vs. less strongly associated concepts (Brunel, 1996; Mongillo et al., 2003). These models can also maintain multiple items in working memory (Haarmann and Usher, 2001; Amit et al., 2003), and can accommodate “overlapping coding,” i.e., the random attribution of the neurons to the coding of the items in memory, whereby some neurons are activated by different concepts coded by different populations (Curti et al., 2004). However, random overlap between populations coding for different concepts cannot contribute to priming because every concept has equal overlap with the others, not allowing a concept to prime some but not all other concepts. One possible solution to this problem is to consider learning the overlap between populations from the relatedness among the concepts they code for, as in recent cortical network models where “mixed-selectivity” neurons are co-active during learning the co-occurrences of related items (Rigotti et al., 2010, 2013; Bourjaily and Miller, 2011; Lavigne et al., 2014). Endowing the Lerner and Shriki model with the ability to learn the semantic associations would take the model even closer to biological realism.

In conclusion, semantic priming is a broad area of research and illustrates how the context in which the given information is processed is key to cognition. The model by Lerner and Shriki is a welcome attempt at reconciling the many facets of semantic priming within a unified (and more biologically plausible) framework, one that resonates with several contemporary ideas of how the brain deals with learning representations for context-dependent decisions.

## Conflict of interest statement

The authors declare that the research was conducted in the absence of any commercial or financial relationships that could be construed as a potential conflict of interest.
